# The C-terminal Domain Supports a Novel Function for CETPI as a New Plasma Lipopolysaccharide-Binding Protein

**DOI:** 10.1038/srep16091

**Published:** 2015-11-05

**Authors:** Victor García-González, Nadia Gutiérrez-Quintanar, Jaime Mas-Oliva

**Affiliations:** 1 Instituto de Fisiología Celular, Universidad Nacional Autónoma de México. 04510 México, D.F. México; 2 Facultad de Medicina, Universidad Autónoma de Baja California, Mexicali, 21000 Baja California, México

## Abstract

Described by our group a few years ago, the cholesteryl-ester transfer protein isoform (CETPI), exclusively expressed in the small intestine and present in human plasma, lacked a functional identification for a role of physiological relevance. Now, this study introduces CETPI as a new protein with the potential capability to recognise, bind and neutralise lipopolysaccharides (LPS). Peptides derived from the C-terminal domain of CETPI showed that CETPI not only might interact with several LPS serotypes but also might displace LPS bound to the surface of cells. Peptide VSAK, derived from the last 18 residues of CETPI, protected against the cytotoxic effect of LPS on macrophages. At high concentrations, when different cell types were tested in culture, it did not exhibit cytotoxicity by itself and it did prevent the expression of pro-inflammatory cytokines as well as the generation of oxidative stress conditions. In a rabbit model of septic shock, the infusion of peptide VSAK exerted a protective effect against the effects of LPS and reduced the presence of tumor necrosis factor-alpha (TNFα) in plasma. Therefore, CETPI is proposed as a new protein with the capability to advance the possibilities for better understanding and treatment of the dangerous effects of LPS *in vivo*.

It has been shown that the cholesteryl-ester transfer protein isoform (CETPI) is only expressed in the small intestine and that it is present in plasma under basal conditions^1^. Unlike the cholesteryl-ester transfer protein (CETP), CETPI lacks exon 16, and 54 bases contained in intron 15 form part of the new mRNA, replacing the 24 C-terminal residues present in CETP with a sequence of 18 residues containing a high concentration of prolines and positively charged amino acids^1^.

Based on phylogenetic studies, a family of proteins known as the PLUNC (palate, lung and nasal epithelium clone) family has been described; it includes proteins associated with immunomodulation, lipid transport and LPS binding. Members of this family include proteins such as CETP, the bactericidal/permeability-increasing protein (BPI)^2^ and the lipopolysaccharide-binding protein (LBP).

Although BPI and LBP both contain the most important structural elements of and show strong three-dimensional structural similarities with CETP, they only present a 19% and 21% homology in primary structure, respectively. In this regard, it is interesting that in terms of primary structure, CETP and CETPI show an exact amino acid sequence with the single exception of 18 residues at their C-terminal regions and therefore show 98% homology^1^. Given the presence of LBP and BPI in human plasma associated with LPS binding activity, this study, exploring the C-terminal domain of CETPI, investigated the potential ability of the newly described protein to bind LPS, as well as its potential interaction with the cell membrane of bacteria and modulation of LPS-induced cellular oxidative stress.

An essential component of the outer membrane of Gram-negative bacteria are LPS, phosphorylated glycolipids unique to each bacterial species. In general, LPS comprise four components: lipid A, the inner core, the outer core and the O-antigen. However, most of the variations found between LPS reside in lipid A, the hydrophobic lipid moiety that anchors LPS to the outer leaflet of the bacteria membrane. Lipid A is also considered an important element since it represents the toxic moiety of these molecules^3^. In the bloodstream, when lipid A is exposed, the immune response is activated and a series of cytokine mediators of inflammation are released into the circulation. In this regard, LPS-mediated dysregulation of the innate immune response following an infection could result in an exacerbated systemic inflammation, leading to hemodynamic pathogenesis and metabolic derangement, which could trigger the development of septic shock^4^,^5^.

It has been reported that the main function of CETP in the reverse cholesterol transport system mobilising cholesteryl-esters from high density lipoproteins (HDL) to very low density lipoproteins (VLDL) rich in triglycerides, is decreased during an infection event and therefore in the development of an inflammatory condition. This phenomenon also leads to a lower synthesis of hepatic CETP mRNA^6^,^7^. Moreover, it has been shown that low serum HDL concentrations might be considered a risk factor in the initiation of septic shock, and even associated with increased mortality^8^. Due to the high serum HDL concentration and the capacity of these lipoproteins to bind LPS, they have been taken as a protective factor against sepsis^8^; the LPS inhibitory action upon CETP in plasma might result in the displacement of the equilibrium towards an adequate formation of HDL particles. An optimal formation of HDL particles would neutralise LPS and effectively direct these particles to the liver. Taking this mechanism into consideration as well as the LPS binding activity of LBP and BPI, our study identifies CETPI as a new molecule involved in the neutralisation of the deleterious effects of LPS.

Based on our experimentation with chemically synthesised peptides derived from the C-terminal domain of CETPI, we propose that the LPS binding properties of this protein reside in this region. In consideration of these results and the three-dimensional structural similarities between CETPI and other LPS binding proteins, we suggest that the new protein discovered in our laboratory has key physicochemical properties that might lead to improved LPS binding characteristics compared to the rest of the members of the PLUNC family. Since CETPI is overexpressed in small intestine cell cultures and has been shown to be present in human plasma^1^, based upon *in vivo* analysis in experimental animals undergoing septic shock, the efficacy of the administration of peptide VSAK with regard to the acute pathological state was studied. The recovery of a normal body temperature and a decrease in the presence of plasma pro-inflammatory molecules such as TNF-α were observed after treatment, suggesting that peptides derived from the C-terminal domain of CETPI and therefore CETPI itself might represent a new group of molecules designed to block LPS action in the circulation^9^. In this context, although many laboratories around the world have recently focused their efforts on the search for a successful therapy that could change the mortality rate caused by this multifactorial disease, the unsuccessful identification of new treatments solely based on the study of hemodynamic phenomena and inflammation as targets has led many researchers to explore apparently distant approaches to the problem^10^,^11^,^12^,^13^. Since the activation of several simultaneous processes contributes to the pathogenesis of sepsis as well as septic shock, and since isolated therapies for each one of these processes have not produced satisfactory results, a new therapeutic paradigm is needed^14^,^15^,^16^,^17^. Thus, this study represents the creation of one of these new concepts, giving CETPI, a recently discovered protein exclusively secreted by the enterocyte, a potential role as a protective barrier against Gram-negative bacteria and against the harmful effects caused by LPS in the circulation.

## Results and Discussion

### Secondary structure of the C-terminal domain of CETPI and CETP

**Figure 1:**
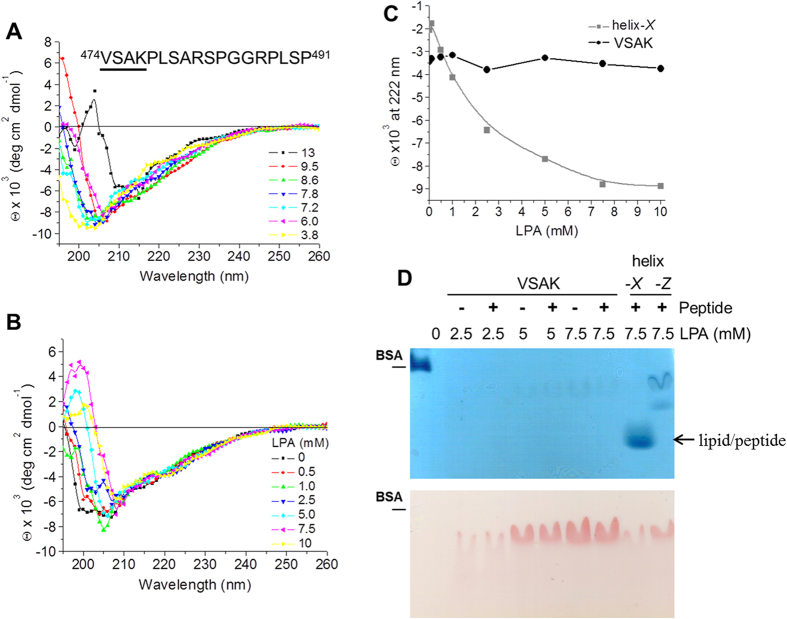
Structural characterisation of CETPI C-terminal domain. (**A**) CD spectra of peptide VSAK obtained at a pH range of 3.8–13. (**B**) CD spectra of peptide VSAK under increasing LPA concentrations. (**C**) Using the same conditions, ellipticity values at 222 nm of peptide VSAK and helix-*X*. (**D**) Non-denaturing polyacrylamide gradient gels (3–40%) of samples processed through cosedimentation assays. Upper panel: Coomassie blue stain. Lower panel: Sudan black stain.

In consideration of the fact that CETPI does not present the key C-terminal α-helix lipid binding domain, but that this domain is present in CETP (helix-*X* domain), we initially performed the structural characterisation of the last 18 residues of the CETPI C-terminus. Circular dichroism (CD) spectroscopy of several C-terminal domain derived peptides indicated that peptide VSAK (V_474_-P_491_) is maintained in a disordered conformation independent of pH-related secondary structural transitions (Fig. 1A), in contrast to the peptides derived from the C-terminal domain of CETP^18^.

Previous work from our laboratory has described conformational changes in the secondary structure of CETP that are dependent on the specific lipid microenvironment associated with the protein. It has been demonstrated that micelles formed with lysophosphatidic acid (LPA)^19^ prepared with peptides derived from the C-terminal domain of CETP show structural transitions coupled with a lipid-ordering phenomenon^20^. In contrast, this type of lipid-dependent structural transition does not occur when CETPI peptides are evaluated (Fig. 1B). These experiments show that the disordered structure present in peptide VSAK is maintained, and no response is produced by the use of increasing LPA concentrations, showing constant ellipticity values at 222 nm (α-helix signal). In contrast, CETP-helix-*X* shows a response associated with the presence of two transitional states (Fig. 1C). These results show a clear difference as to the way in which secondary structural transitions at the C-terminal domain of CETP and CETPI are regulated by a lipid-related microenvironment.

Following a methodology developed in our laboratory for the characterisation of peptide-lipid interactions that employs cosedimentation assays and peptide bond spectroscopy^20^, we demonstrated that peptide VSAK derived from CETPI does not interact with lipids such as LPA. Non-denaturing polyacrylamide gel electrophoresis confirmed that lipid binding did not occur when peptide VSAK was evaluated (Fig. 1D). Compared to CETP, the CETPI C-terminal domain peptide VSAK is a less flexible sequence and has a positive electrostatic charge, hydrophilic characteristics and prolines; in general it shows secondary structural disorder and points to an entirely new non-lipid-related function for CETPI.

Sequence analysis of the CETPI C-terminal domain indicates that in a condition close to neutral pH, the net electrostatic charge for peptide ARS is +2 and for peptide VSAK it is +3, maintained employing a broad pH range (Supplementary Fig. 1). An increase in the size of the CETPI-derived peptides allows positive electrostatic charges to decrease, as is the case of the 27-residue peptide called INPE (I_465_-P_491_) with a charge of +1, and that of peptides EII (29 aa/E_463_-P_491_), SKG (36 aa/S_456_-P_491_) and SRL (48 aa/S_444_-P_491_) where the net electrostatic charge corresponds to 0. In this regard, the highest net positive charge is specifically associated with peptide VSAK, containing the last 18 residues (Supplementary Fig. 1). Disordered secondary structures such as the CETPI C-terminal region, which shows a high positive isoelectric point, could represent an important property that facilitates the interaction of a localised region of the protein with negatively charged surfaces. Since structural rigidity generally hinders activity^21^, in agreement with several authors studying antimicrobial peptides and proteins^22^,^23^, our results are consistent with the possibility that CETPI might perform an important function against Gram-negative bacteria or molecules derived therefrom; for instance, by binding to their membrane and/or neutralising *in vivo* the dangerous effects of free circulating LPS.

### Functional characterisation of peptides

Based on protocols in which PAGE electrophoresis was used for the study of LPS lipid A and O-antigen^24^, we developed a protocol employing native polyacrylamide gradient gels to characterise LPS-peptide interactions.

**Figure 2:**
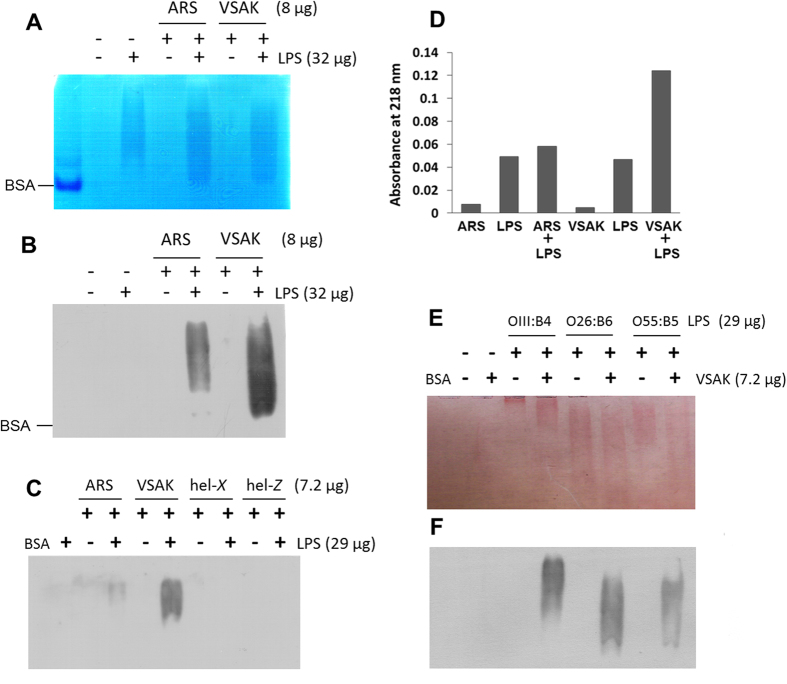
LPS-binding properties of CETPI C-terminal domain. (**A**) Electrophoresis in native polyacrylamide gradient gels (0.8–25%) stained with Coomassie blue employing LPS, peptides ARS, VSAK and mixtures of LPS and peptide. (**B**) Western blot analysis employing the anti-CETPI A481-P491 antibody that recognizes the C-terminus domain. (**C**) Analysis of peptide-LPS binding detected when peptides ARS, VSAK, hel-*X* and hel-*Z* are incubated with LPS serotype 0111:B4. (**D**) Peptide bond absorbance of pellet samples obtained after cosedimentation experiments. (**E**) LPS-peptide VSAK binding identified employing serotypes 0111:B4, O26:B6, O55:B5 and evaluated by Sudan black staining, and (**F**) by western blot analysis using the anti-CETPI antibody.

When LPS are subjected to electrophoresis, it is common to observe a smearing pattern instead of well-defined bands^24^. Under our experimental conditions, when the CETPI C-terminal peptides were subjected to electrophoresis, no signal bands were observed (Fig. 2A). Nevertheless, peptides previously incubated with LPS were retained in the gels as shown by Western blot analysis (Fig. 2B). When the experiment was performed in the presence of peptides helix-*X* and helix-*Z*, both derived from CETP, LPS were not able to bind to these peptides and therefore were not retained in the gel matrix (Fig. 2C). These results reflect the strong interaction achieved between CETPI-derived peptides and LPS. This observation is supported by peptide bond spectroscopy done after the cosedimentation experiments, in which peptide VSAK was principally detected in samples incubated with LPS (Fig. 2D).

Additional experiments for analysis of LPS binding specificity were conducted using three *E. coli* LPS serotypes. Peptide VSAK indistinctly presented LPS binding properties to O111:B4, O26:B6 and O55:B5 serotypes, confirmed by a smearing pattern observed when Sudan black stain was used (Fig. 2E). Western blot analysis of this experiment showed that the anti-CETPI antibody A481-P491 is able to recognise samples when LPS of a different serotype are incubated with peptide VSAK (Fig. 2F).

**Figure 3:**
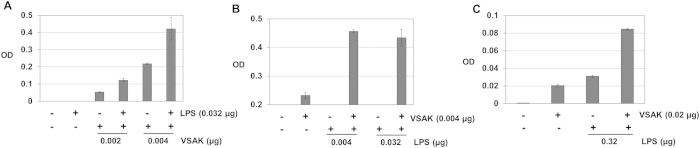
Characterisation by ELISA of LPS-peptide VSAK binding. (**A**) ELISA plates coupled with LPS maintaining constant peptide VSAK concentration. (**B**) Constant peptide VSAK concentration and LPS concentration increments. (**C**) ELISA plates coupled with peptide VSAK incubated with LPS analyzed employing an anti-lipid A antibody (26–5). (Mean values, n = 6, X ± S.E.M.).

An ELISA detection system was also developed by our group in order to broaden the peptide VSAK-LPS interaction^9^. When LPS (0.032 μg) were attached to the plate surface, a signal increase in optical density (OD) was identified only in samples incubated with peptide VSAK (0.004 μg), as an indication that the binding process was taking place (Fig. 3A). Employing a constant concentration of peptide VSAK, a signal increase in OD was exclusively shown in samples incubated with LPS under a concentration range of 0.004–0.032 μg (Fig. 3B). In supplementary experimentation using an anti-lipid A IgG, peptide VSAK attached to the plate surface was identified only in the presence of LPS (Fig. 3C). In summary, this experimental evidence demonstrates the LPS binding capabilities of peptide VSAK.

**Figure 4:**
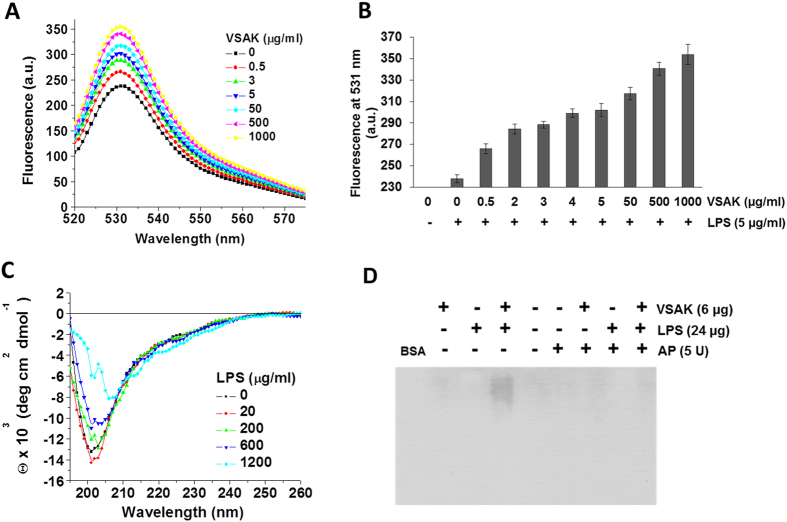
Peptide VSAK promotes a rearrangement phenomenon on LPS structure. (**A**) Peptide VSAK treated with the LPS-BODIPY FL probe. Emission spectra employing a 520–575 nm range were registered using an excitation wavelength of 513 nm. (**B**) Fluorescence emission values at 531 nm (maximum value) under increasing peptide VSAK concentrations. (**C**) Peptide VSAK (200 μg/ml) secondary structure analysis evaluated by CD after incubation with LPS. (**D**) Alkaline phosphatase dephosphorylation effect on LPS-peptide VSAK binding, evaluated by Western blot analysis.

The use of 5 μg/ml of the fluorescent conjugated LPS (LPS-BODIPY FL) showed an enhancement in fluorescence emission spectra under incubation with gradually increasing peptide VSAK concentrations (Fig. 4A), where the highest emission spectrum was recorded at 531 nm. An evident increase in emission fluorescence for peptide VSAK was recorded from 50 μg/ml to the highest concentration of 1000 μg/ml (Fig. 4B). Increases in fluorescence value in the presence of LPS have been also described in experiments employing BPI and LBP; this phenomenon has been associated with an optimal exposure of specific binding sites for LPS^25^. Further, when the secondary structure of peptide VSAK was evaluated in the presence of LPS, we did not find changes in secondary structure and for this reason the peptides remained in a disordered state (Fig. 4C).

Several studies have shown that the biological effects of LPS can be reduced if negative charges in the phosphate groups of lipid A are modified^26^. In this respect, alkaline phosphatase (AP) dephosphorylation experiments were conducted. Our results show that after treatment, a decrease in LPS binding to peptide VSAK was observed (Fig. 4D). Since several authors have proposed that a cluster of positive residues at the tip of the N-domain of LBP corresponds to a critical site for LPS interaction^27^,^28^, it is interesting to note that peptide VSAK has peptide characteristics similar to those of the LBP N-domain. Our results suggest that the LPS binding properties of peptide VSAK may be determined by electrostatic interactions at the lipid surface, and considering that peptide VSAK covers the last 18 residues of the CETPI C-terminus, residues K_477_, R_482_ and R_487_ may be critical to this interaction (Supplementary Fig. 1). Therefore, the binding of peptide VSAK to LPS may be related to the potential neutralisation of negative electrostatic charges of phosphate groups located in lipid A. Since several of the most important driving forces for peptide adsorption onto lipid membranes are hydrophobicity, electrostatic and hydrogen bonding^29^, the electrostatic nature of the LPS binding activity of peptide VSAK was also evaluated under a range of ionic strength conditions. By Western blot analysis employing NaCl concentrations between 200 mM and 1.4 M, our results demonstrate a decrease in binding with direct correlation to an increase in ionic strength (Supplementary Fig. 2A). Under the same conditions, when binding was evaluated employing LPS-BODIPY FL, a significant reduction in fluorescence at 531 nm was also recorded (Supplementary Fig. 2B).

### Cell culture experimentation

**Figure 5:**
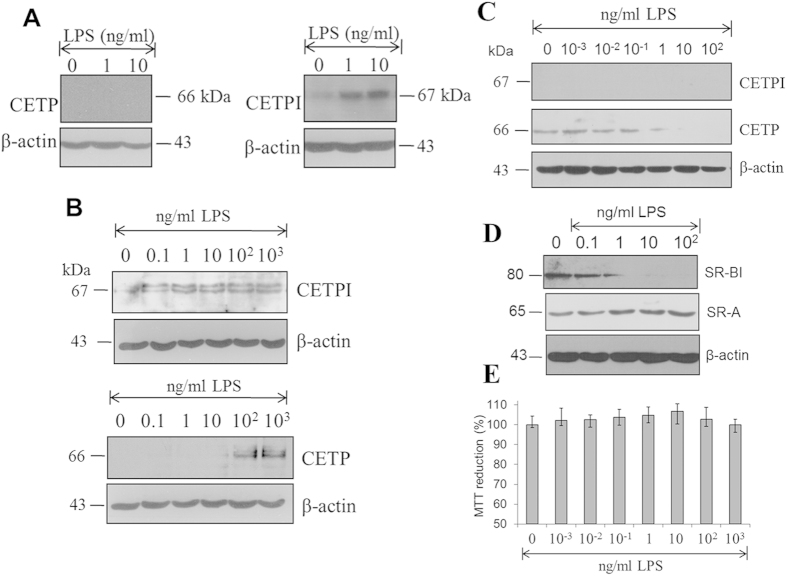
Differential expression of CETPI and CETP under a LPS stimulus. (**A**) CETP and CETPI expression in Caco2 cells under LPS treatment. Specific antibodies for each protein respective C-terminal domains were employed. (**B**) CETPI and CETP expression in small intestine FHs74Int cells after 12 h treatment of gradual increased LPS concentrations. (**C**) CETPI and CETP expression in HepG2 cells treated with LPS. (**D**) Expression of SR-BI and SR-A in HepG2 cells under treatment with increasing concentrations of LPS. (**E**) Cell viability measured by MTT under the same experimental conditions. β-actin used as loading control (Mean values, n = 6, X ± S.E.M.).

With the objective of broadening the LPS-CETPI relationship from a cellular perspective, protein expression of colon-derived cells (Caco-2) was evaluated under the stimuli of various LPS concentrations. Although an increase in CETPI expression was found secondary to an LPS stimuli (1 and 10 ng/ml), CETP synthesis in this cell type was not detected (Fig. 5A). CETPI expression in small intestine cell (FHs74Int) cultures was also evaluated. When these cells were treated with various LPS concentrations, an increase in CETPI expression was detected (Fig. 5B). Interestingly, in the evaluation of the expression of CETP under the same conditions, only LPS concentrations above 10^2^ ng/ml switch on CETP synthesis (Fig. 5B). In accordance with information previously reported by our group^1^, CETPI synthesis with or without LPS treatment was non-existent in the hepatocyte. In contrast, CETP normally synthesised by the hepatocyte was inhibited by high concentrations of LPS when it was added to the culture medium (Fig. 5C). Remarkably, under increasing LPS concentrations, analysis of expression of SR-BI and SR-A reflects important differences, since SR-A expression seems to slightly increase, while SR-BI seems to decrease in response to the presence of LPS in the medium (Fig. 5D). This response to LPS stimulation in the expression of scavenger receptors in the hepatocyte may be associated with a compensatory response of hepatocytes to ameliorate the cytotoxic effects of LPS, and also associated with changes in CETPI expression, as a defence mechanism designed to prevent inflammation and synthesis of pro-inflammatory molecules and acute phase proteins in the liver.

Consequently, in order to discard the possibility that a decrease in CETP and SR-BI synthesis could be associated with changes in HepG2 cell viability, MTT assays were performed. No significant differences in viability were found with treatment, regardless of LPS concentration (Fig. 5E). Thus, the differences shown between the expression of CETP and CETPI in colon cells, small intestine cells and hepatocytes treated with LPS give us an idea of their well-differentiated *in vivo* functions despite their notable structural similarities.

CETP and CETPI most probably originate from the same single gene where alternative RNA splicing or DNA arrangement explain the structural differences between them. Nevertheless, if we take into consideration that CETP, for example, is apparently capable of performing two functions; cholesteryl-ester transfer activity and LPS binding capability at high LPS concentrations, then the one gene encoding for CETP presents two entirely different functions, through gene sharing, and therefore can be considered a moonlighting protein^30^. The same situation applies to CETPI, where in this case the protein has been mainly designed for LPS sequestration, most probably maintaining the property of binding neutral lipids.

Joram Piatigorsky and Graeme J. Wistow, with regard to their work with crystallins, a family of moonlight proteins, concluded that “such a double constraint could significantly slow the evolutionary clock for parts of these genes while elsewhere leading to accelerated change in the selection of modifications beneficial to the more recent function”^30^. This could be just one of the reasons why from the evolutionary point of view, CETP, a protein that short circuits the removal of cholesteryl-esters and which is mainly considered a deleterious protein, is still present in human plasma.

**Figure 6:**
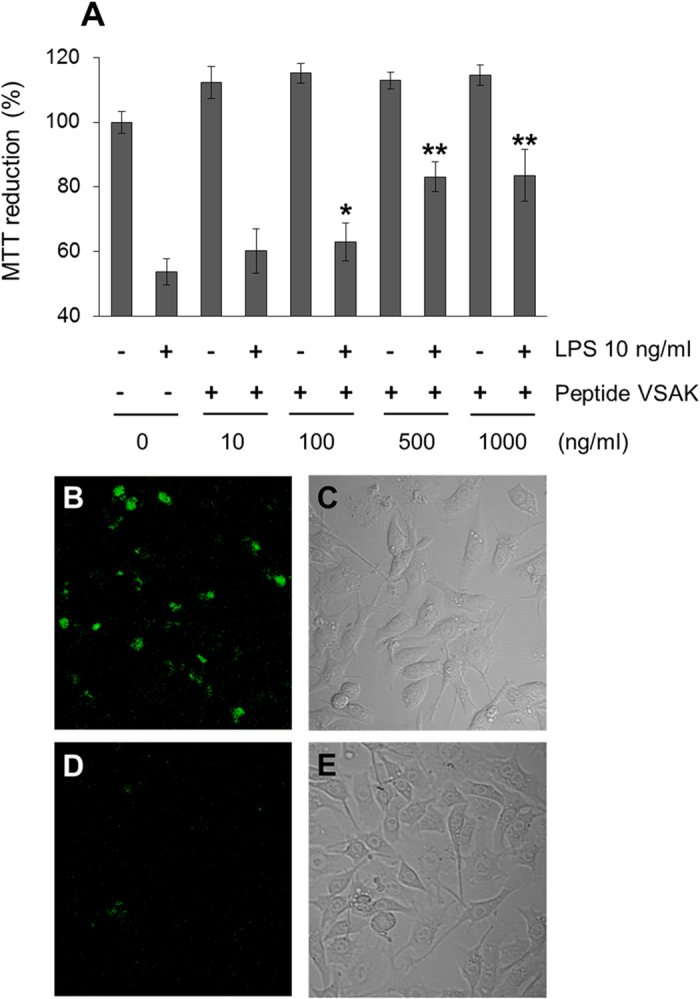
Peptide VSAK prevents cytotoxic effects in macrophages. (**A**) Cell viability evaluated by MTT of macrophages treated for 45 min with increasing doses of peptide VSAK and LPS 10 ng/ml. Mean values are presented (n = 6, X ± S.E.M.), *p < 0.05, **p < 0.001 compared to control group. (**B**) Confocal microscopy and (**C**) light microscopy of macrophages stimulated with LPS-BODIPY (0.5 μg/ml) for 4 h. (**D**) Confocal microscopy and (**E**) light microscopy of macrophages treated with peptide VSAK (50 μg/ml) under the same LPS-BODIPY conditions.

Taking into account that macrophages represent a well-defined cellular model for the study of LPS biology^31^, RAW 264.7 cells were incubated for 45 min with increasing concentrations of peptide VSAK and were further stimulated with LPS (10 ng/ml). While the MTT assays showed that LPS treatment induced a 50% decrease in cell viability, the addition of peptide VSAK into the cell culture gradually reduced this effect (Fig. 6A). The highest protective effect was observed with VSAK at 1000 ng/ml, representing a lipid-to-peptide ratio of 1:100. Under these conditions, there were no cytotoxic effects produced by VSAK itself (Fig. 6A). The use of the probe LPS-BODIPY FL with RAW 264.7 cells confirmed the presence of an evident effect of peptide VSAK on LPS internalisation under the same lipid-peptide ratio (Fig. 6B–E).

In order to test the ability of peptide VSAK to protect macrophages against the deleterious effect of LPS serotype 0111:B4, a set of experiments was designed in which, to cells previously treated with LPS (10 ng/ml) in culture for 0, 2, 4 and 12 h, 3 different concentrations of peptide VSAK were added for an additional incubation of 20 h (Supplementary Fig. 3). LPS 0111:B4 has been characterised as a serotype that activates an intracellular response leading to the activation of inflammatory caspase-11^5^,^32^,^33^. It is interesting to observe that, dependent on time, although the viability effect of LPS upon macrophages is important, treatment with peptide VSAK allows viability values to recover close to values produced in control experiments. From these experiments it becomes clear that peptide VSAK not only exerts a protective effect on macrophage viability (Fig. 6), but also may be binding and probably displacing LPS bound to the plasma membrane of cells (Supplementary Fig. 3).

**Figure 7:**
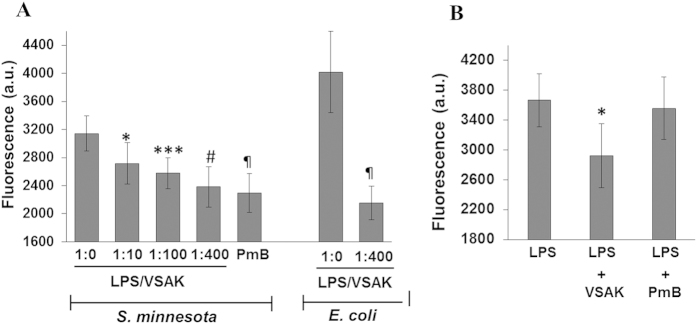
Peptide VSAK incubation interferes with LPS binding to macrophage receptors. (**A**) Inhibition of LPS binding to the cell surface by peptide VSAK incubation. *S. minnesota* and *E. coli* 055:B5 LPS, both coupled to Alexa probe were used. PmB used as a control. (**B**) LPS displacement from the cell membrane by peptide VSAK. Mean values are presented (n = 5, X ± S.E.M.), *p < 0.1, ***p < 0.01, #p < 0.005, ^¶^p < 0.001 compared to control groups.

In a standardised macrophage lipoprotein binding assay^34^, the presence of peptide VSAK in the culture medium induced a decrease in the amount of LPS-Alexa Fluor that binds to the macrophage cell surface when a lipid-peptide ratio of 1;10, 1;100 and 1;400 is evaluated in parallel with standard polymyxin B (PmB)-treated cells. Polymyxin B, an antibiotic primarily used against Gram-negative bacteria, has been shown to alter bacterial outer membrane permeability and to inactivate LPS^35^. Data obtained with a ratio of 1;400 using LPS from *S. minnesota*, showed an effect similar to that of PmB in the reduction of LPS bound to the cell membrane. This response was also produced when LPS from *E. coli* were tested (Fig. 7A). These results suggest that peptide VSAK binds to LPS in solution, thereby blocking the interaction of LPS with the cell surface. With the purpose of continuing to test the possibility that peptide VSAK is apparently able to displace LPS already bound to the cell surface, macrophages incubated with LPS for 2 h to which peptide VSAK was added showed a decrease in LPS-Alexa Fluor associated with the cell membrane (Fig. 7B). Interestingly, although polymyxyn B displays the ability to bind LPS in solution, it is apparently incapable of displacing LPS already bound to the cell membrane (Fig. 7B). It was also shown that an LPS stimulus on macrophages can induce the formation of reactive oxygen species (ROS), a phenomenon that is decreased in the presence of peptide VSAK (Supplementary Fig. 4A). With the use of confocal microscopy, the protective effect of peptide VSAK against the promotion of oxidative stress by LPS was clearly observed (Supplementary Fig. 4B–E).

We have previously shown that macrophages under oxidative stress induce changes in the expression of several adaptor proteins important to the organisation of the endocytic machinery^36^. In this respect, we found that there is a diminished expression of proteins such as the endocytic adaptor β-adaptin in an oxidative stress event^18^,^36^. In that study, this result was secondary to the establishment of oxidative stress, and related to an impaired internalisation of ligands due to the lack of organisation of the endocytic machinery^36^,^37^,^38^. New experiments carried out in our laboratory have now shown that endocytic proteins such as β-adaptin and amphiphysin are able to bind to transcription factors such as c-myc; this process is related to the control of the cell cycle and affected by oxidative stress (personal communication).

**Figure 8:**
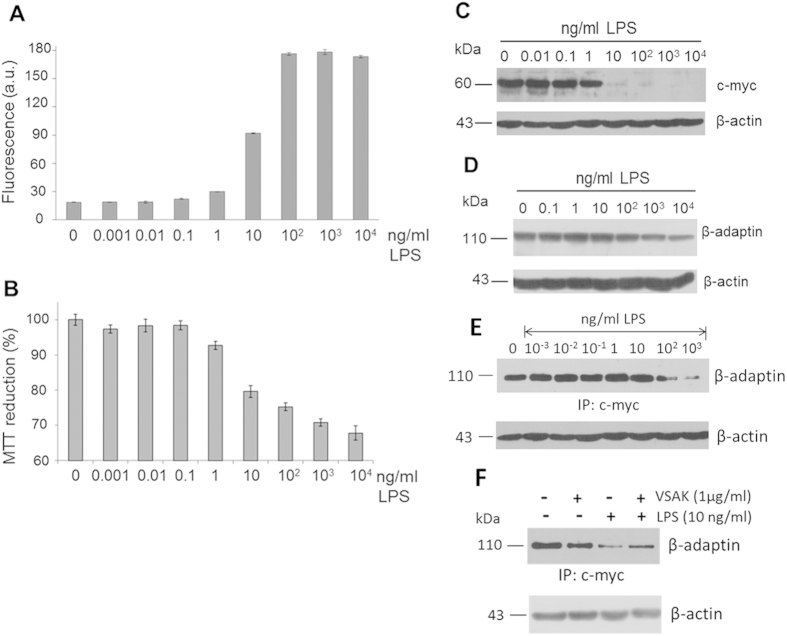
Reactive oxygen species induced by LPS stimuli promote a decrease in c-myc and β-adaptin expression. (**A**) Flow cytometry measurements were performed for the detection of ROS in macrophages treated with increasing amounts of LPS for 12 h. (**B**) MTT viability assays. (**C**) Western blot detection of c-myc and (**D**) β-adaptin. (**E**) Evaluation of the interaction among c-myc and β-adaptin under treatment with increasing concentrations of LPS. (**F**) Western blot of an immunoprecipitation experiment of c-myc from macrophages incubated with LPS (10 ng/ml) and peptide VSAK (1 μg/ml). In all cases β-actin used as control.

In order to determine whether or not the establishment of an oxidative stress event secondary to the presence of LPS in the RAW 264.7 cell incubation medium might also interfere with the organisation of the endocytic machinery associated with a transcription factor such as c-myc, a series of assays was set up. First, we found that an increase in fluorescence associated with the presence of ROS in the RAW 264.7 cell culture medium was directly related to the presence of LPS in the medium (Fig. 8A). Viability assays performed in parallel showed that a concentration of LPS of 10 ng/ml can be considered critical since at this concentration the degree of cell damage starts to become important (Fig. 8B).

When the same cells were used and the presence of β-adaptin was studied by Western blot (WB) analysis, the critical concentration of LPS (10 ng/ml) dramatically affected the presence of c-myc (Fig. 8C) and β-adaptin (Fig. 8D) in the nucleus of these macrophages. Following immunoprecipitation assays of the c-myc present in the nucleus associated with β-adaptin in LPS-treated macrophages, we determined that LPS promotes a decrease in the interaction between c-myc and β-adaptin, possibly as an early response mechanism to cell damage (Fig. 8E). Moreover, at the critical concentration of 10 ng/ml, it was found that although LPS treatment decreased the amount of the adaptor protein β-adaptin in the nucleus, the presence of peptide VSAK in the cell incubation medium tended to restore the concentration of nuclear β-adaptin and in consequence its interaction with c-myc (Fig. 8F).

These results show for the first time that the deleterious effect of LPS on ROS production might be directly linked to a disarray of the cytoskeleton and the synthesis of transcription factors such as c-myc associated with control of the cell cycle. It is interesting to note that when peptide VSAK blocks the action of LPS, it restores the normal sequence of events that controls the synthesis of β-adaptin and c-myc.

For many years, the recognition of bacterial LPS as components of the cell membrane of Gram-negative bacteria performed by the innate immune system was thought to involve simple pathways, as compared to the adaptive immune system. Today this view has dramatically changed since complex pathways involving multiple receptors and protein-protein interactions have been described^39^. It has been suggested that the activity of LPS is effected through its high-affinity binding to LBP. The LPS/LBP complex carries out its action by associating with the CD14-toll like receptor located at the surface of monocytes and macrophages^40^,^41^,^42^. The activation of these cell types promotes the release of a network of proinflammatory molecules such as TNF-α, interleukins (IL-1α, IL-1β, IL-6, IL-8 and IL-12) and other inflammatory molecules such as platelet-activating factor^43^,^44^,^45^. All of these factors are directly involved in several well-known uncontrolled inflammatory responses from a localised area, all the way to the systemic level. This chain of events could lead to multiple organ failure and a life-threatening condition known as septic shock^46^, an exacerbated systemic reaction to an infection, which is currently recognised as one of the most common causes of death in intensive care units worldwide^47^,^48^. Despite the efforts made to counteract what is known as inflammatory disequilibrium syndrome which is in general the outcome of septic shock, current therapeutic possibilities, mainly involving control of inflammation and metabolism with antibiotic treatment, have not changed for many years. For this reason, important attempts have been made to find new treatments and new molecules that can handle and treat sepsis and its consequent septic shock. In this direction, antimicrobial peptides (AMPs), mostly showing positively charged and amphipathic characteristics, have been recently described as antimicrobial agents for clinical purposes^49^,^50^. AMPs are synthesised by most living organisms and are considered part of their immune system. They carry out their antimicrobial action by forming pores and disrupting the normal flux of nutrients and ions across the membrane of a large number of microorganisms^51^,^52^. These characteristics have also been reported to be the basis for several of the secondary effects and toxic events described when AMPs have been used in the hospital setting^53^,^54^.

Since there are reports that state that HDL particles are able to bind LPS and that propose therefore that they also work as a protective factor against sepsis^8^, we aimed to determine whether or not the presence of peptide VSAK could somehow influence the way in which LPS bind to human HDL. These lipoproteins, when incubated with LPS, coupled to Alexa Flour 488 fluorescent probe, and further ultracentrifuged through a KBr gradient to remove unbound probe, showed slight differences in the amount of LPS bound by the presence in the incubation medium of increasing concentrations of peptide VSAK (Supplementary Fig. 5). Taking into account the apparently high binding ability of peptide VSAK for LPS observed in experiments shown above and the slight displacement of labelled LPS from HDL only shown by the highest peptide concentrations used in this set of experiments, might allow us to speculate that LPS binding to HDL presents a high affinity and therefore the presence of CETPI in plasma or the infusion of peptide VSAK in the circulation of patients in septicaemia or septic shock would not displace LPS already bound to HDL particles.

### *In vivo* experimentation

**Figure 9:**
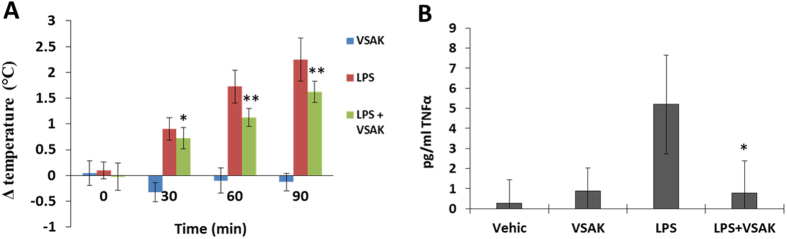
Peptide VSAK blockade of LPS action in a septic shock model established in rabbits. (**A**) Rectal temperature measurements throughout 90 min. Normalized values from temperatures that ranged from 37.5 °C to 40.0 °C. Mean values are presented (n = 5, X ± S.E.M.), *p < 0.05, **p < 0.01 obtained in the analysis of the LPS group with respect to the LPS plus peptide VSAK group. (**B**) Serum TNFα values obtained at the end of the 90 min experiment. Mean values are presented (n = 5, X ± S.E.M.).

In order to test the potential use of peptide VSAK as a blockade molecule against the *in vivo* action of LPS, a pilot experiment was designed employing rabbits as the experimental animal model. Using four groups with five experimental animals each, our results showed no increase in rectal temperature for 90 min with 30 min measurement intervals in the two groups of control animals injected in the marginal ear vein with vehicle or peptide VSAK alone (Fig. 9A). In contrast, when LPS 0111:B4 were injected (0.3 μg/kg) using the same route of administration, an immediate increase in temperature was recorded after 30 min, with an increase from 37.5 °C to 40 °C after 90 min treatment. However, when peptide VSAK was injected into the marginal vein of the right ear immediately followed by an injection of LPS 0111:B4 (0.3 μg/kg) in the marginal vein of the left ear, the increase in temperature was less pronounced throughout the 90 min duration of the experiment. The amount of peptide VSAK injected corresponds to an LPS-peptide VSAK ratio of 1;200, the same molecule ratio used in our *in vitro* cellular experiments. The supplementary measurement of cytokine TNFα in the serum of the experimental animals at the end of the 90 min body temperature measurements indicated that TNFα was not found under control conditions, while a significant quantity was measured in the group treated only with LPS 0111:B4. Again, the group of animals that received the infusion of peptide VSAK 5 min before the lipopolysaccharide injection showed a decrease in plasma TNFα levels (Fig. 9B).

Considering the nature of the LPS-peptide VSAK interaction, it is interesting to observe that peptide VSAK by itself did not show any secondary effects and that it did exert a protective effect in the group of experimental animals further injected with LPS. Although peptide VSAK under our experimental conditions does not show bactericidal activity (data not shown), its function related to the blockade of LPS action contrasts with several AMPs that do not present lipid specificity and do induce secondary toxic effects^55^. Experiments performed with several cell lines (Caco-2, HepG2 and microglial EOC) previously reported to be very sensitive to toxic stimuli^36^ showed that peptide VSAK does not cause any effects deleterious to viability when used at high concentrations (data not shown), which indicates its high degree of safety.

In consideration of the fact that a high LPS concentration has been reported in the environment surrounding the intestinal epithelium in several pathological conditions^56^,^57^, design made by nature through evolutionary processes most probably permitted, on the one hand, the existence of CETPI in order to control LPS cytotoxicity close to the enterocyte and in plasma, and on the other, the existence of CETP as a key protein in the homeostasis of lipoprotein cholesterol, by only slightly modifying their generic protein structure at the carboxy-end region. Although for some time now there has been the goal to design and use peptides as therapeutic molecules to inactivate LPS^58^,^59^,^60^, CETPI emerges as a protein present in human plasma that might prove to be important inactivating LPS in circulation.

Since CETPI that is synthesised by intestine cells and found in the circulation shares a 98% structural homology with CETP, it would not be unreasonable to think that molecules such as torcetrapib with CETP as a target, might also be binding to CETPI and therefore impeding the proper recognition and binding of LPS by this newly described protein. This possibility emerges from the fact that torcetrapib, used in the recent past to block the function of CETP in the fight against the development of atherosclerosis, had to be withdrawn from advanced clinical trials due, amongst other complications, to the fact that treatment resulted in an increased ratio of infections compared to control groups^61^.

In this context, peptides such as VSAK that form part of the carboxy-end sequence of CETPI, emerge as potential agents for the neutralisation of LPS in the circulation, and therefore as a new generation of molecules in the therapeutic arsenal against the negative action of LPS in septicemia and septic shock^9^,^62^. As recently put forward by Professor Tom van der Poll, “We need to redouble our efforts to identify new targets for therapeutic intervention and refine our clinical research methods to translate research findings to new treatments for severe sepsis”^63^.

## Materials and Methods

### Materials

L-α-Phosphatidylcholine (PC), 1-oleoyl-2-hydroxy-sn-glycero-3-phosphate (LPA), 1-lauroyl-2-hydroxy-sn-glycero-3-phosphocholine (lyso-C_12_PC) and 1-palmitoyl-2-oleoyl-sn-glycero-3-phospho-1-glycerol were obtained from Avanti Polar Lipids. Cholesterol, MTT, LPS serotypes (O111:B4, O26:B6, O55:B5) and PmB were purchased from Sigma-Aldrich. Fluorescent conjugates of LPS BODIPY FL and LPS Alexa-Fluor 488 from *E. coli* O55:B5, LPS Alexa Fluor 488 from *Salmonella minnesota*, and 6-carboxy-2′,7′-dichlorodihydrofluorescein diacetate were obtained from Invitrogen. Antibodies used in the immunoprecipitation assays and anti-lipid A (26–5) antibody were from Santa Cruz Biotechnology. Alkaline phosphatase (AP) was purchased from New England BioLabs.

### Peptide synthesis and preparation

Considering the amino acid sequence and physicochemical properties of the CETPI carboxy-end segment, several peptides were synthesised. Peptide VSAK corresponds to the last 18 residues (V_474_-P_491_), peptide ARS (A_481_-P_491_) to the last 11 residues, while peptide INPE (I_465_-P_491_) corresponds to the last 27 residues (Supplementary Fig. 2). Lyophilised peptides were dissolved at 1 mg/ml and a further 1:5 dilution was carried out. The effect of pH on secondary peptide structure was evaluated in a pH range of 3.8–13, and the characterisation of lipids was evaluated only at pH 7.2.

Two peptides derived from the CETP C-terminal domain were used as a control: native peptide helix-*X* (E_465_-S_476_), and helix-*Z* (E_465_-S_476_) containing the D_470_N mutation (Supplementary Fig. 2). Peptide purity greater than 98% was confirmed by mass spectrometry and HPLC analysis (GenScript).

### Circular dichroism (CD) spectroscopy

CD spectra were recorded with an AVIV 62DS spectropolarimeter (AVIV Instruments) at 25 °C employing far UV wavelengths (190–260 nm). Experiments were conducted at a peptide concentration of 200 μg/ml in a 1 mm quartz path length cuvette using AVIV software. Spectra were recorded with a 1 mm bandwidth, using 1 nm increments and 2.5 s accumulation time. CD results are reported as mean molar ellipticity (*Θ*, deg cm^2^ dmol^−1^) considering the baseline correction.

### *In silico* analysis

In order to analyse the three-dimensional structure of CETP and BPI, the STRAP algorithm (Interactive Structure based Sequences Alignment Program) was used. Structures were obtained from the protein data bank (PDB), with access codes 2obd and 1bp1 for CETP and BPI, respectively.

### CETP and CETPI antibodies

CETP and CETPI antibodies that specifically recognise their respective C-terminal domains were obtained from chemically synthesised peptides corresponding to the last 11 residues of their C-terminal region. In order to allow coupling with Keyhole Limpet Hemocyanin, peptides included a cysteine at the amino-end. Peptides coupled to KLH were used in the production of anti-CETP IgY and anti-CETPI IgY employing white Leghorn chickens and using a standard protocol of 63 days as described by Alpha Diagnostic International. Antibody titres were determined by ELISA assays. Protocols used were based on methodology employed by our group in the development of related patent applications^64^,^65^,^66^.

The conditions used for the anti-CETPI A481-P491 antibody during the ELISA assays were established as previously described^9^. Using this technique it was possible to devise a method for the quantification of CETPI and create the conditions for the development of a diagnostic kit^9^.

### Lipid/peptide cosedimentation assays and peptide bond spectroscopy

Cosedimentation experiments were conducted using ultracentrifugation with an Optima TLX ultracentrifuge fitted with a TLA-100.2 rotor as described previously^20^. Supernatants were recovered and the remaining pellets were resuspended in a phosphate buffer with a pH of 7.2. Then, peptide bond absorbance of supernatants and pellets was measured at 205 and 218 nm.

### Polyacrylamide gradient gel electrophoresis

LPA and lyso-C_12_PC samples were processed using 3–35, 3–38 and 3–40% non-denaturing polyacrylamide gels^20^. Gradient gels were stained with Coomassie blue G-250 and Sudan black according to previous work^20^. For LPS sample characterisation, we established a new methodology through the use of 0.8–25% gradient gel electrophoresis. This method allows for the study of lipid-peptide association under native conditions without modifying the properties of the molecules^9^.

The influence of ionic strength on peptide VSAK-LPS binding was also evaluated using increasing concentrations of NaCl by gradient gel electrophoresis and WB analysis. The same type of experiment was evaluated after AP treatment and also through LPS-BODIPY FL fluorescence.

### Particles formed by diverse lipid mixtures and LPS

PC and cholesterol particles were prepared with a molar ratio of PC 2 mM and cholesterol 0.1 mM (20:1). Lipids were mixed in chloroform and dried for 2 h under a gentle stream of N_2_, with an additional period of 12 h in a Speed Vac concentrator (Savant). After drying, lipids were resuspended in PBS and subsequently sonicated with 4 cycles of 10 min (15 s on/30 s off pulses) in an ice bath under an N_2_ flow. Samples were left to equilibrate for 2 h and then centrifuged for 10 min at 13,000 rpm. For lipid polar extracts from *E. coli* (2 mg/ml), we employed the same methodology with 5 cycles of sonication. LPS were dissolved in ultrapure water (1 mg/ml) and sonicated in a water bath for 10 min. Micelles formed by lysophosphatidylcholine and lysophosphatidic acid were prepared following methodology that we had previously designed^20^.

### Cell cultures

FHs74Int (ATCC) epithelial small intestinal cell cultures were grown in a Hybri-Care medium supplemented with 10% fetal bovine serum (FBS) and 30 ng/ml of epidermal growth factor. Caco-2 cells from colon (ATCC) were maintained in MEM medium with the addition of 20% FBS. RAW 264.7 cells (ATCC, macrophages) were grown in RPMI 1640 medium with 10% FBS. HepG2 cells (ATCC) were grown in MEM medium with 10% FBS and sodium pyruvate 1 mM. Penicillin (50 U/ml) and streptomycin (50 μg/ml) were added to the media. Cell viability determination under the different conditions used was evaluated through the MTT reduction assay, based on our previous work^18^.

Macrophage cell cultures placed in 96-well plates (14,000 cells/well) at 80% confluence were treated for 45 min with increasing peptide VSAK concentrations, and were further cultured for 24 h in the presence of LPS (10 ng/ml). After treatment, cell viability was measured through the MTT assay^18^. In another set of experiments, macrophages received 10 ng/ml LPS stimuli for 2, 4 and 12 h, prior to the addition of gradually increasing concentrations of peptide VSAK. These experiments were prolonged for an additional 20 h and MTT assays were performed according to protocols used previously^18^.

Macrophages placed in black 96-well plates (Santa Cruz Biotechnology) were incubated for 2 h in serum-free medium, washed twice with PBS and maintained at 4 °C for 30 min. The cells were subsequently incubated with peptide VSAK or PmB for 30 min. LPS-Alexa Fluor was added followed by further incubation for 2 h at 4 °C. Two washes with PBS were performed and fluorescence measurements were taken with a Synergy HT (BioTek) microplate reader, using an excitation wavelength of 495 nm, an emission of 519 nm and a gain setting of 120.

The peptide VSAK-LPS binding was characterised in the supernatant of the culture medium. The RAW cells were treated with peptide VSAK for 45 min before the LPS-BODIPY FL was added in a proportion of 1 LPS molecule per 100 molecules of peptide VSAK, and incubation continued for 4 h. Extracellular media were recovered and concentrated using a centricon step. Fluorescence was monitored using an Olis spectrofluorimeter and samples were analysed by gradient gel electrophoresis (0.8–25%) and visualised using a Typhoon 9400 device.

Macrophages placed on black 96-well plates were incubated for 1 h in serum-free medium, before being treated with peptide VSAK (10 μg/ml) for 45 min. An LPS stimulus (100 ng/ml) was added and maintained for 4 h at 37 °C. At the end of this assessment period, the fluorescent probe 6-carboxy-2′,7′-dichlorodihydrofluorescein diacetate (11 μM) was added to the culture medium followed by further incubation for 45 min. Cells were washed and then fluorescence was measured in a Biotek Synergy-HT microplate reader.

### Western blot analysis and immunoprecipitation assays

With a plate confluence of 90%, Caco-2, FHs74Int and HepG2 cells were treated under different lipid conditions for 16 h. Afterwards, cells were lysed for 45 min at 4 °C, and samples were processed according to protocols used in a previous work^18^. For CETP and CETPI detection, primary antibodies anti-CETP IgY and anti-CETPI IgY were used following protocols that we had established previously^9^,^64^,^65^,^66^.

Macrophages were treated with peptide VSAK (1 μg/ml) and LPS (10 ng/ml) for 4 h. Nuclei separation was carried out using a buffer containing sucrose (250 mM)/imidazole (3 mM) pH 7.4 supplemented with protease and phosphatase inhibitors. Cells were scraped from culture dishes and 21 passages were performed through a 22G syringe. For recovery of nuclei, lysates were centrifuged at 3400 rpm for 15 min. The two fractions (supernatant and pellet) were lysed for 25 min at 4 °C, and both fractions (20 μg/lane) were analysed by SDS-PAGE and transferred to PVDF membranes. For c-myc detection, the rabbit polyclonal anti-c-myc (1:600) was used, and for β-adaptin a goat polyclonal anti-β-adaptin (1:3000) was used. Membranes were incubated with primary antibodies and after successive washes membranes were incubated with their respective secondary antibodies (1:5000) and horseradish peroxidase activity was detected.

For the immunoprecipitation assays, cytoplasm (400 μg) and nucleus (300 μg) fractions were incubated with an anti-c-myc antibody (1:400) for 2 h at 4 °C. Immune complexes were precipitated with Protein G agarose Fast Flow (Millipore) ON at 4 °C. Immunoprecipitated proteins were washed 3 times and resuspended in Laemmli buffer, separated by SDS-PAGE gels and transferred to PVDF membranes for WB analysis. Detection of β-adaptin was performed based on previous protocols^18^.

### Fluorescence assays

LPS (O55:B5) coupled to BODIPY FL and Alexa Fluor 488 probes were evaluated under the effect of peptide VSAK incubation. Fluorescence emission spectra were recorded at 25 °C from 520 to 575 nm. An excitation wavelength of 503 nm was used for LPS-BODIPY FL, and 519 nm for LPS-Alexa Fluor. LPS-peptide mixtures were incubated for 3 h at 30 °C and readings were taken using an Olis DM45 spectrofluorometer.

### Alkaline phosphatase (AP) assays

LPS O111:B4 (24 μg) were treated with AP (5 U) for 1 h at 37 °C. AP activity was inactivated with a 5 min step at 70 °C. Peptide VSAK (6 μg) was added to dephosphorylated LPS, and further incubated at 25 °C. Treated samples were evaluated by gradient gel electrophoresis (0.8–25%) and WB analysis.

### Confocal microscopy

A confocal scanning biological microscope FV1000 (Olympus) was employed in order to study the cellular localisation of LPS-BODIPY FL and the effect of treatment with peptide VSAK. After 3 h of treatment, cell images were obtained with the use of an excitation wavelength of 503 nm. In addition, ROS localisation in cells was done using the 6-carboxy-2′,7′-dichlorodihydrofluorescein diacetate probe.

### LPS binding to HDL particles

Isolation of the HDL fraction from human plasma (density 1.063–1.21 gKBr/ml) was performed according to established methods and based on a protocol described by Toledo-Ibelles *et al.*^67^. Samples were quantified by the bicinchoninic acid method (BCA) and HDL labelling was performed with 1,1′-dioctadecyl-3,3,3′3′-tetramethylindocarbocyanine perchlorate (Dil) (Molecular Probes). Binding assays were conducted by preparing samples using a constant LPS concentration (7 μg/ml) and coupling to LPS probes Alexa Fluor 488 and BODIPY FL, and 400 μg/ml of HDL. The effect of peptide VSAK was studied employing 7, 35, 700 and 1400 μg/ml. Mixtures were incubated in PBS for 3 h at 37 °C and mixed with a magnetic stirrer at 40 rpm. After incubation, the HDL fraction was again centrifuged to remove free LPS molecules and the samples quantitated by the BCA method. Readings were performed with a microplate spectrofluorimeter (BioTek Synergy/HT).

### Experimental animal protocol

Male New Zealand rabbits (2 kg) were maintained in a temperature- and humidity-controlled location, under *ad libitum* feeding conditions and with free access to water. Four experimental groups with three experimental animals each were evaluated: group one was administered vehicle solution. Group 2 was administered peptide VSAK (60 μg/kg) and group 3 was administered LPS (0.3 μg/kg). Group 4 was administered peptide VSAK and LPS. 24 h fasting animals were injected with treatments in the marginal vein of the ear and every 30 min the rectal temperature was recorded.

Ear blood samples were obtained from animals post-experimentation and serum was recovered and stored in aliquots at −70 °C. For the quantitative measurement of TNFα, an ELISA Kit was employed according to the instructions of the manufacturer (Cloud-Clone Corp). After 90 min, animals were sacrificed via sodium pentobarbital administration.

### Ethics statement

The animal experiment was approved by the Animal Care Committee at Instituto de Fisiología Celular, Universidad Nacional Autónoma de México (JMO22–14). All animal experimentation was performed in accordance to the Declaration of Helsinki and international guiding principles in the care and use of experimental animals under the Mexican Official Norm for Laboratory Animals (NOM062ZOO1999).

## Additional Information

**How to cite this article**: García-González, V. *et al.* The C-terminal Domain Supports a Novel Function for CETPI as a New Plasma Lipopolysaccharide-Binding Protein. *Sci. Rep.* **5**, 16091; doi: 10.1038/srep16091 (2015).

## Supplementary Material

Supplementary Figures
