# Supplementary material for: The C-terminal Domain Supports a Novel Function for CETPI as a New Plasma Lipopolysaccharide-Binding Protein
Source: Sci Rep. 2015 Nov 5;5:16091. doi: 10.1038/srep16091 (PMC4633601; doi:10.1038/srep16091)
Supplement: Supplementary Figures [file srep16091-s1.doc]

**The C-terminal Domain Supports a Novel Function for CETPI as a New Plasma Lipopolysaccharide-Binding Protein.**

**Victor García-González, Nadia Gutiérrez-Quintanar and Jaime Mas-Oliva.**

**Supplementary Fig. 1. Sequence analysis of 48 terminal domain residues of CETPI**. (A) Amino acid sequences of several peptides derived from the C-terminal domain; residues with a positive charge (green), negative charge (yellow). (B) Electrostatic charge distribution of peptides under different pH conditions. Helix-*X* was used as a reference.

**
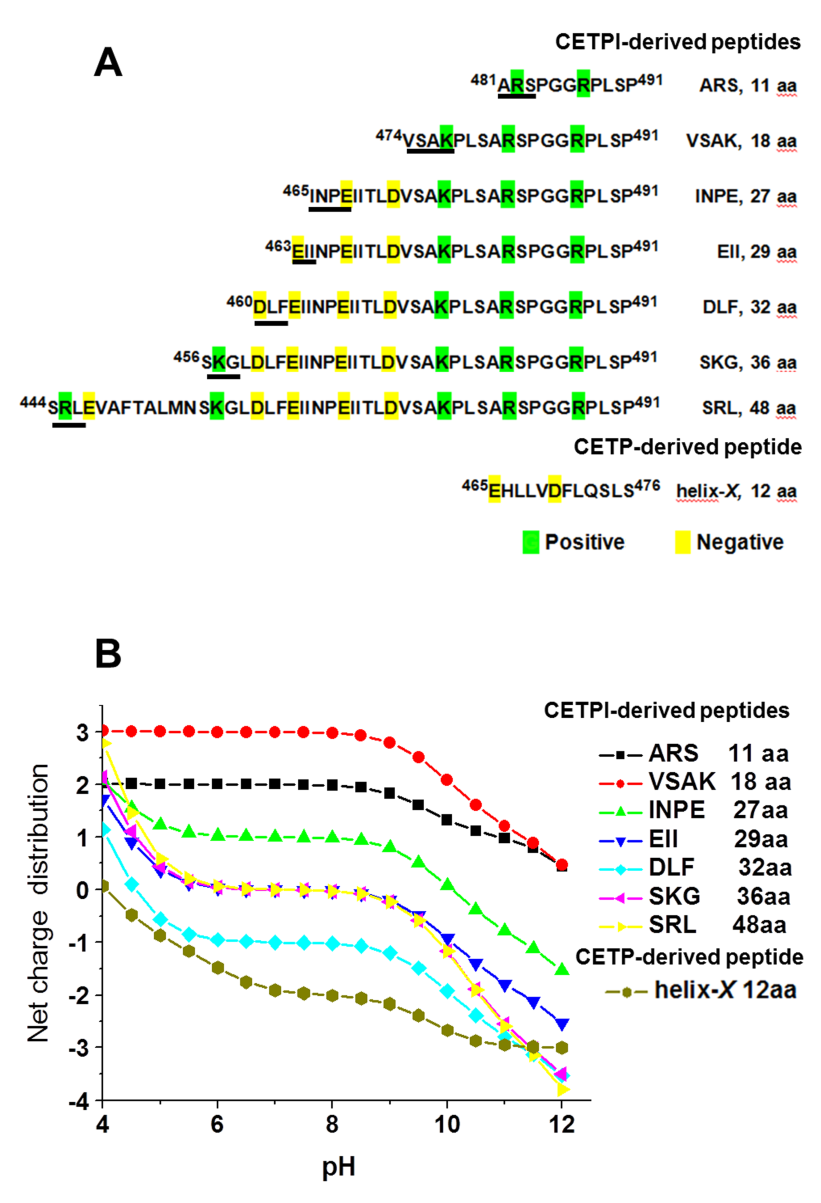
**

**Supplementary Fig. 2. LPS-VSAK binding is defined by electrostatic interactions**. (A) Effect of ionic strength on LPS-VSAK binding evaluated by increasing NaCl concentrations in the incubation medium and visualized by Western blot analysis on native polyacrylamide gradient gels. (B) Emission fluorescence values registered at 531 nm (Mean values, n=6, X ± S.E.M.).

**
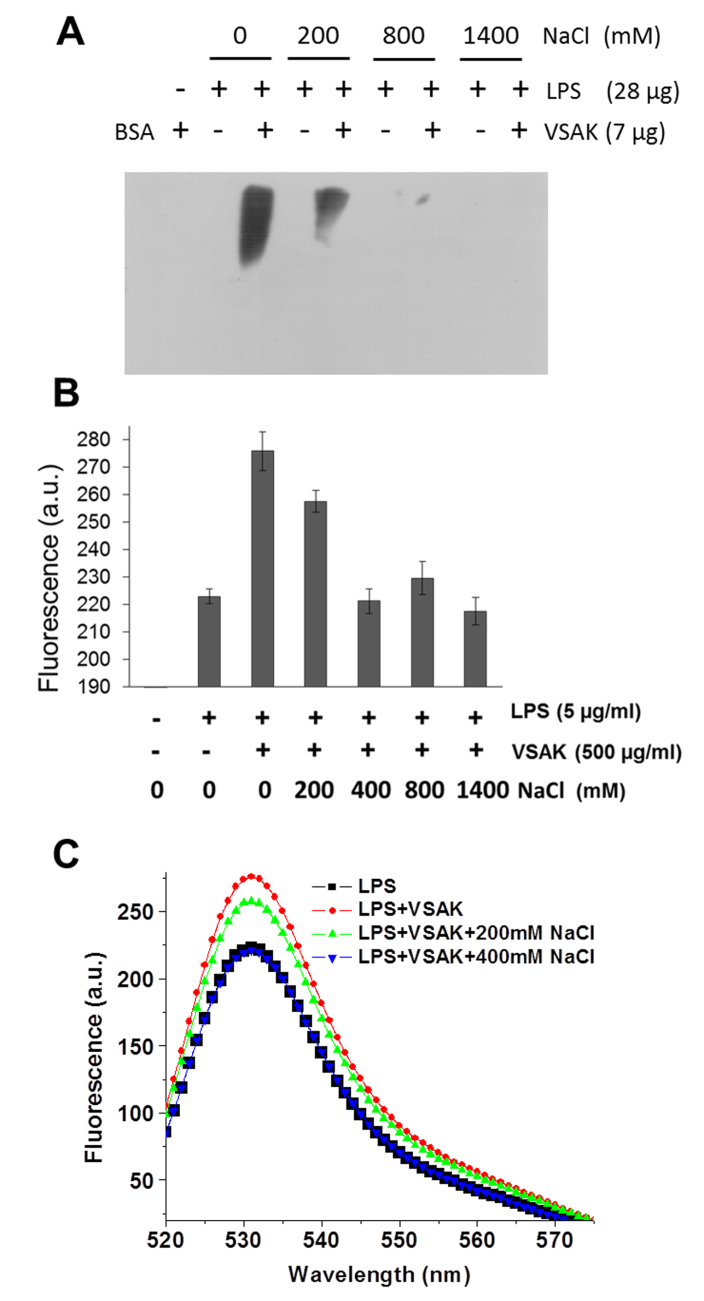
**

**Supplementary Fig. 3. VSAK treatment protects macrophages against LPS cytotoxicity.** (A) Cell viability control experiment incubating peptide VSAK 45 min before a LPSO111:B4 (10 ng/ml) stimulus. (B) Cell viability obtained after LPS treatment 2 h previous the addition of peptide VSAK. (C) LPS treatment 4 h previous the addition of peptide VSAK. (D) LPS treatment 12 h previous the addition of peptide VSAK. In all conditions after peptide VSAK administration, cells were incubated for 20 h. Mean values are presented (n=6, X ± S.E.M.), *p˂0.05, **p˂0.01, #p˂0.001, ¶p˂0.0001 compared to control groups.

**
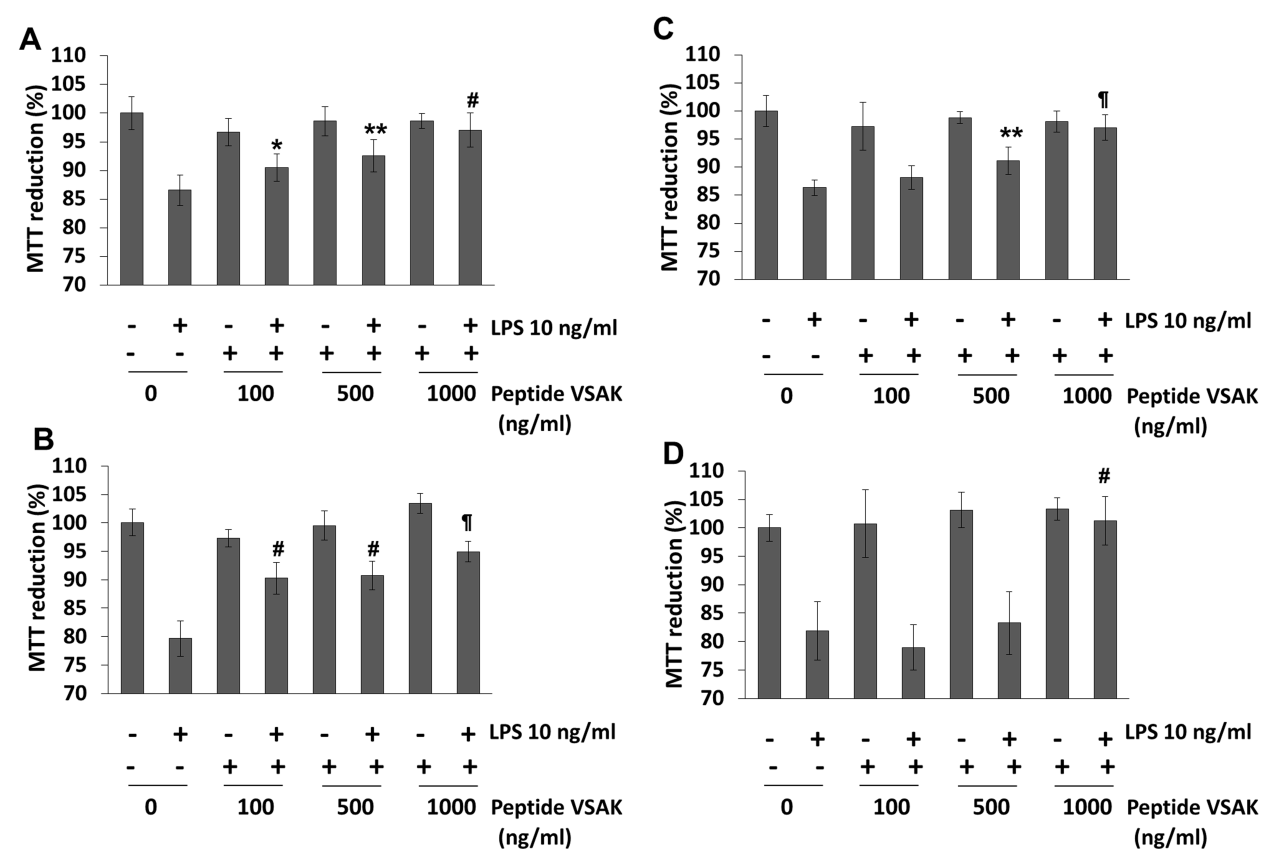
**

**Supplementary Fig. 4. VSAK treatment attenuates macrophage ROS production caused by LPS.** (A) ROS production evaluated using dihydrofluorescein diacetate. Mean values are presented (n=6, X ± S.E.M.), *p˂0.05 compared to control group (LPS+VSAK). (B) ROS identification by confocal microscopy of cells treated for 4 h with LPS (100 ng/ml). (C) Light microscopy as in B. (D) ROS identification by confocal microscopy of cells treated with LPS (100 ng/ml) in the presence of peptide VSAK (10 µg/ml). (E) Light microscopy as in D.

**
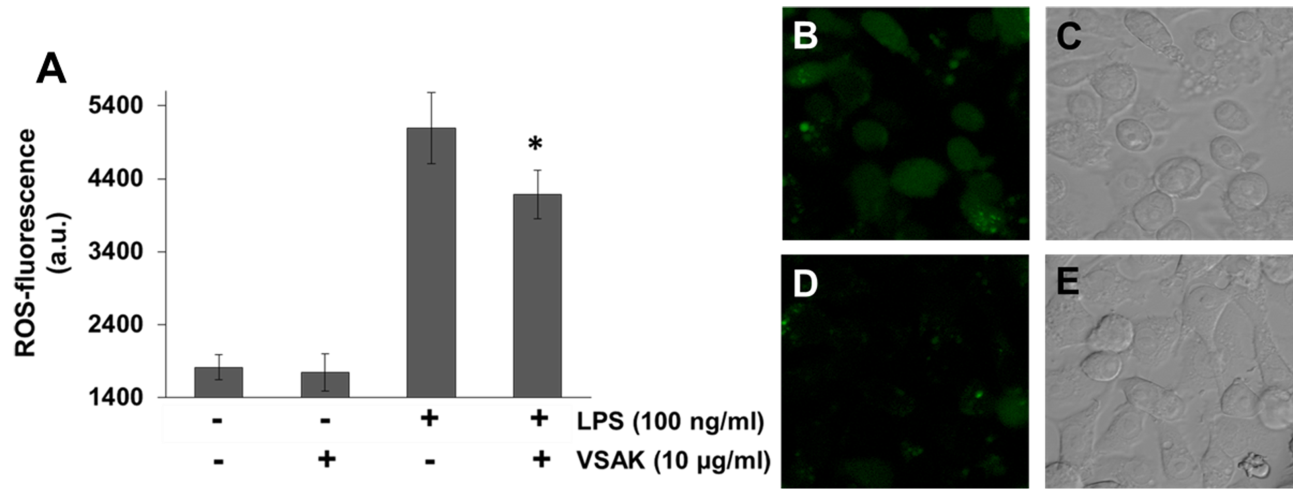
**

**Supplementary Fig. 5. Effect of peptide VSAK on LPS binding to HDL**. A) Purified HDL fluorescence values associated with the LPS-Alexa probe after incubation for 3h at 37° C. B) Fluorescence emission spectra employing a 510-560 nm range under the same conditions. Mean values are presented, *p˂0.5 compared to the HDL+LPS group. Two independent experimental panels were performed.

**
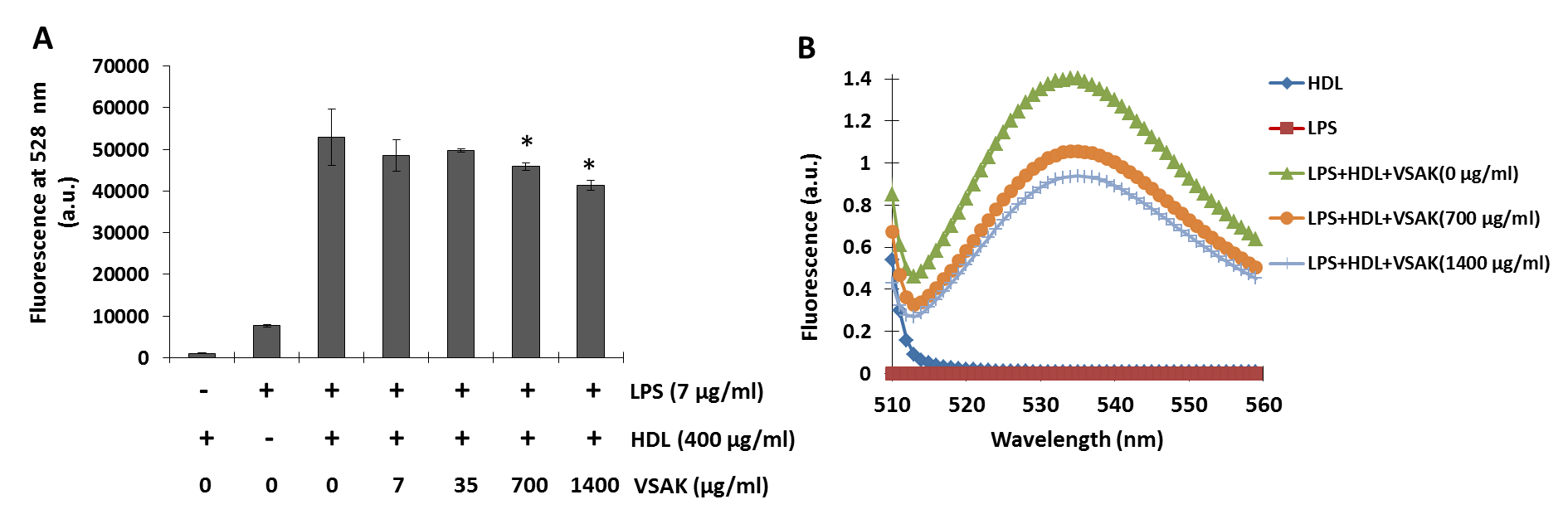
**
